# Waste-to-Energy: Production of Fuel Gases from Plastic Wastes

**DOI:** 10.3390/polym13213672

**Published:** 2021-10-25

**Authors:** Cheuk-Fai Chow, Chow-Shing Lam, Kai-Chung Lau, Cheng-Bin Gong

**Affiliations:** 1 Department of Science and Environmental Studies, The Education University of Hong Kong, 10 Lo Ping Road, Tai Po, Hong Kong, China; 2 Department of Chemistry, City University of Hong Kong, Kowloon Tong, Hong Kong, China; chowslam@cityu.edu.hk (C.-S.L.); kaichung@cityu.edu.hk (K.-C.L.); 3 The Key Laboratory of Applied Chemistry of Chongqing Municipality, College of Chemistry and Chemical Engineering, Southwest University, Chongqing 400715, China; gongcbtq@swu.edu.cn

**Keywords:** ball-milling, polymer degradation, C-H bond activation, hydrogen atom transfer

## Abstract

A new mechanochemical method was developed to convert polymer wastes, polyethylene (PE), polypropylene (PP), and polyvinyl chloride (PVC), to fuel gases (H_2_, CH_4_, and CO) under ball-milling with KMnO_4_ at room temperature. By using various solid-state characterizations (XPS, SEM, EDS, FTIR, and NMR), and density functional theory calculations, it was found that the activation followed the hydrogen atom transfer (HAT) mechanism. Two metal oxidant molecules were found to abstract two separate hydrogen atoms from the α–CH and β–CH units of substrates, [–_β_CH_2_–_α_CH(R)–]*_n_*, where R = H in PE, R = _γ_CH_3_ in PP, and R = Cl in PVC, resulting in a di-radical, [–_β_CH^•^–_α_C^•^(R)–]. Subsequently, the two unpaired electrons of the di-radical were recombined into an alkene intermediate, [–_β_CH = _α_C(R)–], which underwent further oxidation to produce H_2_, CH_4_, and CO gases.

## 1. Introduction

The great demand for plastics in modern society and the huge amounts of unrecycled plastic waste produced worldwide pollute our environment. In 2019, the amount of plastic wastes produced worldwide every year reached 368 million metric tons [1]. Eight billion polyethylene (PE) shopping bags are disposed of in landfill each year [1]. The handling of these several hundred million tons of “extra-long-lasting” wastes annually is currently an unsolved problem because we still do not have economical, high-efficiency technologies for plastic waste management. The understanding of the activation of these inert polymers could lead to utilization of these hydrocarbon resources, making use of the enormous amounts of hydrocarbon wastes. Unfortunately, the current recycling efficiency of these materials is not as good as required. For example, in 2018, approximately 75.5% of plastic materials (27.0 million tons) were sent to landfill in the USA [2], while in the EU, approximately 14.9% of plastic wastes (7.2 million tons) were sent to landfill [1]. Although alternative strategies for the conversion of unrecycled plastic wastes into fuel or useful materials are in continuous development, including photolysis [3], pyrolysis [4], ultrasound [5], biodegradation [6], chemolysis [7], ozonolysis [8], and catalytic degradation [9], they involve non-selective oxidation and are prone to creating secondary pollution, such as the emission of polycyclic aromatic hydrocarbons and/or dioxin. Given these factors, developing new methods to convert plastic into useful chemical resources, such as fuel gases, organic fuels and/or fine chemicals, in a rapid, mild, and cost-effective way, is important.

Mechanochemical degradation of polymers was first investigated approximately 50 years ago [10]. According to the IUPAC Compendium of Chemical Terminology, a mechanochemical reaction is defined as a “chemical reaction that is induced by the direct absorption of mechanical energy” [11]. Mechanical energy, such as that arising from friction, impacts, and collisions, can offer certain advantages [12,13], as such processes are simple, solvent-free, and effective for various chemical reactions and processes including the formation of carbon–carbon [14] and carbon–heteroatom bonds [15], redox reactions by solid oxidants [16] and reductants [17], and cross dehydrogenative coupling reactions [18]. In addition, mechanochemistry is gaining attention as it has been demonstrated to be applicable in various important scientific areas, such as catalysts [19], nanoparticles [20], organic synthesis [21], MOFs [22], oxides [23], biomaterials [24], supramolecular chemistry [25], and process engineering [26]. In recent years, some attempts have been made to apply this technology to plastic waste management [27] because plastic wastes can be broken down under mechanical force such as agitation, grinding, or extrusion [28,29,30,31,32,33,34,35,36]. Polyvinyl chloride (PVC) was reported to be partially degraded, showing a decrease in molecular weight, formation of C=C bonds in its structure, and production of chlorine ion, when subjected to a mechanochemical process in the presence of metal hydroxides or metal oxides [31,32]. In another study, after undergoing a mechanochemical process by ball-milling with CaO and Ni (OH)_2_ followed by pyrolysis, PVC was able to generate hydrogen, carbon monoxide, carbon dioxide, and methane gas as fuels. This waste-to-energy mechanochemical process targeting PVC wastes is very attractive, but it requires high temperatures (450 °C) and is a multi-step process [35].

Thus, we herein report the C–H activation of solid PE, polypropylene (PP), and PVC to yield fuel gases including hydrogen, methane, and carbon monoxide by using a high-valent manganese oxidant (KMnO_4_) under ball-milling [36] mechanochemical reaction conditions. We found that the first step of the interaction between KMnO_4_ and the α–CH unit of the polymers, i.e., [–_β_CH_2_–_α_CH(R)–] *_n_*, where R = H in PE, R = _γ_CH_3_ in PP, and R = Cl in PVC, results in the homolytic cleavage of the α–CH unit via hydrogen atom transfer (HAT). Through density functional theory (DFT) calculations, we found that the intermediate generated after the first step, [(KMn^VI^O_3_(OH)(–_β_CH_2_–_α_C^•^(R)–)], dissociates into a free radical, [–_β_CH_2_–_α_C^•^(R)–], leading to a second HAT between a new molecule of KMnO_4_ and the _β_–CH_2_ unit to form a di-radical, [–_β_C^•^H–_α_C^•^(R)–]. Subsequently, the two unpaired electrons of the di-radical are recombined into an alkene entity [–_β_CH = _α_C(R)–]. In the presence of sufficient KMnO_4_, more alkane radicals were formed; hence hydrogen, methane, and carbon monoxide were eventually produced. This protocol was subsequently applied in the conversion of real plastic waste samples, including HDPE bottles, PP microwave containers, and PVC water pipes into fuel gases. These findings differ significantly from the previously reported chemolysis of PVC with KMnO_4_ conducted in the liquid phase, as chemolysis results in only the partial dechlorination of PVC [37]. We also note that, recently, one study demonstrated the solvent-free oxidative dehydrogenation of a semi-conjugated substrate, γ-terpinene, to a fully conjugated product, *p*-cymene, using KMnO_4_ via ball-milling [16].

## 2. Materials and Methods

### 2.1. Materials and General Procedure

PE (average *M*_w_ ≈ 35,000 and average *M*_n_ ≈ 7700), PP (average *M*_w_ ≈ 12,000 and average *M*_n_ ≈ 5000), PVC (average *M*_w_ ≈ 43,000 and average *M*_n_ ≈ 22,000), KMnO_4_, KHSO_4_, and Al_2_(SO_4_)_3_ were purchased from Sigma–Aldrich (HKSAR). The ball-milling process was performed at 25°C in a vertical planetary ball mill (DECO-PBM-V-0.4L) using different sizes of hardened steel balls with diameters of 3, 5, and 10 mm and a sealed airtight stainless-steel chamber of 108 cm^3^ in volume.

Infrared spectra in the range of 500–4000 cm^−1^ were recorded on a Perkin Elmer Frontier FTIR spectrometer. An Agilent 7890B gas chromatograph coupled with a thermal conductivity detector (TCD) was used to perform the gas analyses. Injections were conducted manually with a sample injection volume of 100 µL. Cl_2_ and CO_2_ gases were quantified with a capillary column (Agilent HP-PLOT Q column (40 µm, 30 m × 0.53 mm i.d.)). Injections were carried out in splitless mode. Argon was used as the carrier gas at a flow rate of 5.0 mL/min. The temperature was set at 40°C for 5 min, the injection temperature was 100°C, and the detector temperature was 250°C. H_2_, CH_4_, and CO gases were quantified using an Agilent J&W CP-Molsieve 5-Å capillary column (10 µm, 30 m × 0.32 mm i.d.). Injections were set as a 4:1 split at 12 mL/min. Argon was used as the carrier gas at a flow rate of 18.0 mL/min. The temperature was set at 60°C for 6 min, the injection temperature was 80°C, and the detector temperature was 150°C. SEM (JEOL-6390) coupled with EDS was used to study the surface morphologies of the oxidized polymer particles. XPS (Kratos Axis Ultra DLD) was used to study the functional groups at the surface of the oxidized polymer particles. All solid-state ^13^C-NMR measurements were performed on a JEOL ECZ500R 500 MHz solid-state NMR spectrometer at a 20 MHz proton resonance frequency, using a typical π/2 pulse length of ~3.2 μs and a receiver dead time of ~13 μs at 25 °C.

### 2.2. Ball-Milling of PE, PP, and PVC Using KMnO_4_

Virgin PE, PP, and PVC powders were crushed to obtain particle sizes in the range of 65–125 μm before use. In a typical experiment, oxidation of the polymer, i.e., PE (0.50 g, 18 mmol), PP (0.50 g, 12 mmol), or PVC (0.50 g, 8 mmol), was performed by adding KMnO_4_ (1.2 molar equivalents: 21.6 mmol for PE; 14.4 mmol for PP; and 9.6 mmol for PVC) with or without KHSO_4_ to an airtight stainless-steel chamber (108 cm^3^) with a mix of stainlesssteel balls (diameters of 3, 5, and 10 mm); the ball-to-powder ratio was 55:1. The reaction was conducted under vacuum at 25 °C. The chamber was evacuated, sealed, and fixed in a planetary ball-milling machine. The ball-milling reaction was conducted at a speed of 800 rpm for 12–48 h. The gases produced were collected and analyzed by GC-TCD using the abovementioned procedure.

The black solid residues were washed three times with deionized water. The total organic/inorganic carbon content of the dissolved aqueous solution was determined using a Shimadzu TOC-L CSH high-sensitivity total organic analyzer. The total chloride content of the dissolved solution was performed by standard titration methods using AgNO_3_ and KCrO_4_ as an indicator.

The solid residues left after washing were manganese oxide and insoluble organic polymers. The oxidation state of manganese was studied by the iodometric titrations. After the insoluble organic polymers were obtained by washing the crude solids in aqua regia to eliminate manganese oxide particles, they were oven-dried at 100°C. The collected organic solids (denoted **PE [ox]_vac_**, **PP[ox]_vac_**, and **PVC[ox]_vac_**) were analyzed by FTIR spectroscopy, solid-state ^13^C-NMR, SEM, and XPS.

### 2.3. DFT Calculations

DFT calculations were performed using the B3LYP [38,39,40] functional and the LanL2DZ basis set [41] for Mn and the 6-311++G(d,p) basis set for non-metal atoms. The optimization and frequency calculations were performed in the gas phase. No solvent effects were considered. For these calculations, octane, 2,4,6-trimethyloctane, and 3,5-dichlorooctane were used to represent PE, PP, and PVC, respectively. The geometries were fully optimized, and the structures were confirmed as true minima based on frequency calculations. None of the reactants, intermediates, or products had imaginary frequencies, whereas the TSs had a single imaginary frequency. The connectivity of the TS structures was confirmed by intrinsic reaction coordinate calculations.

## 3. Results and Discussion

### 3.1. Oxidation of Plastic Wastes by Solid-State Ball-Milling

**Scheme 1:**
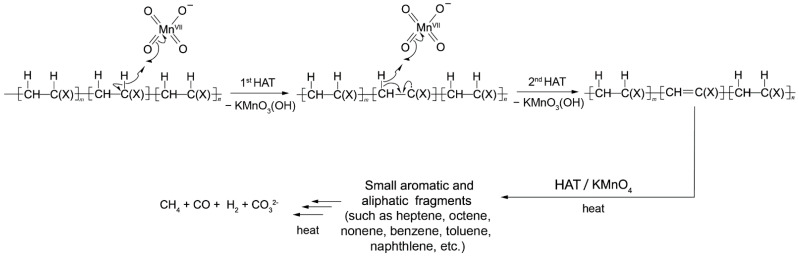
Double HAT mechanism for desaturation: C–H bond activation of PE, PP, and PVC by two molecules of KMnO_4_ through the ball-milling reaction {[–_β_CH_2_–_α_CH(R)–]*_n_*, where R = H in PE, R = _γ_CH_3_ in PP, and R = Cl in PVC}.

In a typical experiment, the solid-state ball milling of polyolefins (PE, PP, and PVC (0.50 g)) with KMnO_4_ (1.2 molar equivalents: 21.6 mmol for PE; 14.4 mmol for PP; and 9.6 mmol for PVC) at a speed of 800 rpm under vacuum for 48 h resulted in a complete decomposition of the polymers. It led to the production of major coal gas components, including gaseous CO, H_2_, and CH_4_. Figure S1 shows the gas chromatography with thermal conductivity detection (GC-TCD) spectra of the gases collected from the reactions. Moderate amounts of H_2_ (3.5–5.6 mmol) and CO (4.6–13.3 mmol) gases were generated, which had retention times of 1.30 and 3.63 min, respectively. A significant amount of methane (1.8 and 4.2 mmol) was also detected with a retention time of 2.41 min in the degradation of PE and PP, whereas only a small amount of methane (0.6 mmol) was produced from the degradation of PVC. The complete dechlorination of PVC was achieved during the ball milling reaction, and a quantitative amount of chloride anions (8.0 mmol) was produced during the process. A large amount of carbon in the polymers was degraded in the form of CO_3_^2^^−^ (6.8–14.5 mmol), and neither CO_2_ nor Cl_2_ gas was detected by GC. Table 1 shows the product yields from the oxidation of the polymer samples. Through iodometric titrations, the manganese residue was determined to be manganese (II) oxide, which could potentially be recycled to KMnO_4_ by chemical/electrochemical oxidation [42]. Various oxidants, including Mn(acetate), Mn_2_O_3_, MnO_2_, K_2_MnO_4_, K_2_Cr_2_O_7_, oxone, PbO_2_, and K_2_S_2_O_8_, were also used to degrade PE, PP, and PVC; however, aside from potassium permanganate, the tested oxidants were ineffective in oxidizing/activating the polymers. Interestingly, Tang and coworkers reported the formation of H_2_, CH_4_, and CO through the catalytic pyrolysis of PP by the formation of alkane radicals with heat (600°C) [43]. Therefore, we initially postulated that the energy released from the polymer degradation, especially in the presence of excess KMnO_4_, could dissociate the polymer into these gases. GC-MS was therefore used to analyze the abovementioned degradation process. Various alkene-based intermediates possessing carbon numbers ranging from 8 to 18 (such as 2,6-dimethyl-3-heptene, 2,2-dimethyl-3-octene, and 4,6,8-trimethyl-1-nonene) and aromatic hydrocarbons with 6–14 carbon atoms (such as benzene, xylene, and phenanthrene) were identified in the extractant of the polymer degradation products. These results confirm that the degradation proceeds via a radical mechanism through which random aromatic and aliphatic hydrocarbons can be formed (Scheme 1) [43,44].

HDPE waste bottles, used PP microwave boxes, and PP water pipes were used to test the applicability of the developed protocol to degrade real polymer waste samples. Upon application of the same mechanochemical process to the polymer waste samples, moderate amounts of CO (4.1–9.2 mmol), H_2_ (2.6–5.0 mmol), and CH_4_ (0.6–4.7 mmol) were generated from the real samples (Table 1). Burning the gases generated from the reaction of the HDPE sample with excess KMnO_4_ resulted in a high-temperature blue flame (see Video S1 in the Supplementary Information). Comparison of the results obtained for the standard polymers and these samples suggested that the mechanochemical prototype was not affected by the presence of additives (dyes or other plasticizers) or dirt in the polymer samples.

### 3.2. Characterization of the Intermediates and Products during Ball-Milling of PE, PP, and PVC with KMnO_4_

The mechanism of the abovementioned reaction was comprehensively investigated by various solid-state spectroscopic characterizations (XPS, SEM, EDS, FTIR, and NMR), and density functional theory calculations. In a typical experiment, the solid-state ball milling of polyolefins (PE, PP, and PVC (0.50 g)) with KMnO_4_ (0.2 molar equivalents: 3.6 mmol for PE; 2.4 mmol for PP; and 1.6 mmol for PVC) and KHSO_4_ (as an acid, 0.2 molar equivalents: 3.6 mmol for PE; 2.4 mmol for PP; and 1.6 mmol for PVC) at a speed of 800 rpm under vacuum for 48 h resulted in brownish-black solid inorganic/organic mixtures. The collected solids were washed with deionized water to give a colorless filtrate, which indicated that the KMnO_4_ had been consumed. The average oxidation states of the manganese residues after the reactions were determined as 3.6 ± 0.01 (PE substrate), 3.8 ± 0.14 (PP substrate), and 4.0 ± 0.09 (PVC substrate), based on iodometric titrations. The appearance of a strong broad infrared (IR) absorbance band at ~560 cm^−1^ indicates the formation of Mn^IV^O_2_ during the reactions. The solid mixtures were then washed with aqua regia to eliminate the inorganic oxide and to yield the oxidized forms of PE, PP, and PVC, denoted **PE[ox]_vac/acid_** (77.9% yield), **PP[ox]_vac/acid_** (85.1% yield), and **PVC[ox]_vac/acid_** (39.7% yield), respectively. In the absence of acid, the corresponding **PE/PP/PVC[ox]_vac_** products were also obtained.

**Figure 1:**
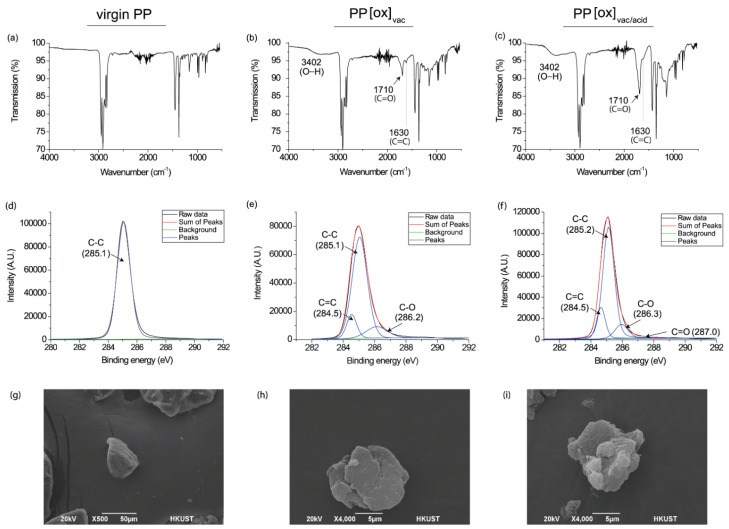
(**a**–**c**) FT-IR, (**d**–**f**) XPS, (**g**–**i**) SEM images of virgin PP, **PP[Ox]_vac_**, and **PP[Ox]_vac/acid_**.

During solid-state ball-milling of solid PE, PP, and PVC by KMnO_4_, the formation of organic intermediates took place. Figure 1a,c shows the FTIR spectra of the virgin PP and **PP[ox]_vac/acid_**. Compared with the spectrum of the virgin state, three new IR absorption peaks were observed in that of **PP[ox]_vac/acid_**, i.e., at 1630, 1720, and 3402 cm^−1^, and these peaks were assigned to the C=C, C=O, and O–H stretching vibrations, respectively [45]. The FTIR spectra of **PE[ox]_vac/acid_** and **PVC[ox]_vac/acid_** also suggested the formation of new functional groups (Figures S2c and S3c). The characterization of the nature of the formed unsaturated groups was conducted by solid-state ^13^C-NMR and X-ray photoelectron spectroscopy (XPS) studies. The ^13^C-NMR spectrum of **PP[ox]_vac/acid_** revealed a peak at ~130 ppm, indicating the presence of non-terminal alkene groups. Furthermore, the ^13^C-NMR spectrum showed a peak at ~225 ppm, indicating the presence of ketone functionalities in the oxidized polymer. In addition, the high-resolution C_1s_ and O_1s_ XPS spectra of **PP[ox]_vac/acid_** indicated the presence of C=C (alkenyl, 284.5 eV), C–C (alkyl, 285.1 eV), C–OH (hydroxyl, 286.3 and 533.6 eV), and C=O (carbonyl, 287.0 and 532.5 eV) groups (Figure 1f). The ratio between the desaturated (C=C) and hydroxylated groups (C–OH and C=O) in **PP[ox]_vac/acid_** was found to be 1.3:1 (Table S1). Furthermore, Figure 1g,i shows typical scanning electron microscopy (SEM) images of the virgin PP and **PP[ox]_vac/acid_**, respectively. The average size of **PP[ox]_vac/acid_** was ~7.5 μm, whereas that of the virgin PP particle was found to be ~105 μm. The surface morphology of **PP[ox]_vac/acid_** was highly irregular. Based on energy-dispersive X-ray spectroscopy (EDS) studies, Cl atoms were found on the surface of **PVC[ox]_vac/acid_**, and chloride anions were also produced during the process. Supplementary Information Figures S2 and S3 show the FTIR spectra, XPS analyses, and SEM images of PE/**PE[ox]_vac/acid_** and PVC/**PVC[ox]_vac/acid_**, respectively.

It is important to note that the production of alkene groups in the polyolefins was enhanced in the presence of acid (KHSO_4_). In comparison with the FTIR spectrum of **PP[ox]_vac/acid_**, a smaller C=C stretching peak at 1600 cm^−1^ was found for **PP[ox]_vac_**. From the XPS analyses, the ratio between the unsaturated and hydroxylated entities in **PP[ox]_vac_** was found to be ~0.8:1 (Figure 1e,f, Supplementary material (Table S1). In addition, in the presence of acid, the desaturated products of PP, PE, and PVC were obtained in 60% higher yields than those obtained in the absence of acid (Table S1).

### 3.3. Reaction Mechanism

Scheme 1 shows the proposed activation and oxidation mechanisms for PE, PP, and PVC induced by ball milling with KMnO_4_. The first step involves the interaction of a KMnO_4_ molecule and an α–CH unit in the polymer, followed by the homolytic cleavage of the α–CH unit via the HAT mechanism, resulting in a KMn(VI)O_3_(OH) and a [–_β_CH_2_–_α_C^•^(R)–] unit. This first HAT is defined as the rate-determining step. Furthermore, we postulated that KMn^VI^O_3_(OH) dissociates from the radical species, [–_β_CH_2_–_α_C^•^(R)–], leading to a second HAT with a second molecule of KMnO_4_ via its –_β_CH_2_ unit, thus forming a di-radical, [–_β_C^•^H–_α_C^•^(R)–]. Finally, the recombination of the two electrons in the di-radical species generates an alkene, [–_β_CH = _α_C(R)–], in the polymer chain. The solvent-free, solid-state ball-milling reaction could therefore facilitate the formation of the di-radical and prevent the rebound mechanism [46].

### 3.4. DFT Calculations

**Figure 2:**
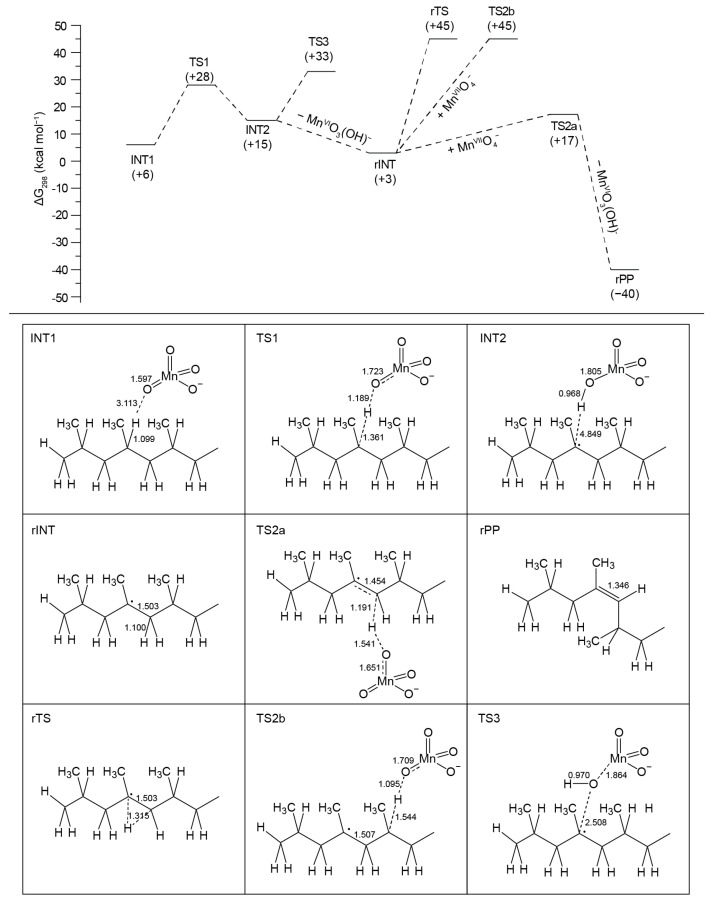
(**Top**) Potential energy surface for the oxidation of PP by MnO_4_^−^ at the B3LYP/LanL2DZ level of theory for Mn and the B3LYP/6-31++G(d,p) level of theory for non-metal atoms. All energies are in kcal/mol and relative to those of the free reactants. (**Bottom**) Structures of intermediates (INTs) and transition states (TSs); the unit of bond distance is Å.

To understand the mechanism further, three sets of DFT calculations were performed for the polymers. More specifically, the PE, PP, and PVC polymer chains were modeled using octane, 2,4,6-trimethyloctane, and 3,5-dichlorooctane, respectively. For simplicity, these prototypical molecules were denoted simply as PE, PP, and PVC. The potential energy surface and the structures of the intermediates (INTs) and transition states (TSs) in the oxidation of PP by KMnO_4_ are shown in Figure 2, whereas those for PE and PP are shown in Schemes S1 and S2, respectively.

Before the first step of the reaction, MnO_4_^−^ forms the van der Waals complex INT1 with a PP chain in an endergonic reaction (Δ*G*_298_ = 6 kcal mol^−1^ relative to the energy of the reactants). The reaction then begins when the oxo ligand from the MnO_4_^−^ ion abstracts an H-atom from the α-position of the PP repeating unit, [–_β_CH_2_–_α_CH(_γ_CH_3_)–], via TS1 (Δ*G*^‡^_298_ = 28 kcal mol^−1^), to form [(Mn^VI^O_3_OH)(–_β_CH_2_–_α_C^•^(_γ_CH_3_)–)]^−^ (INT2). The dissociation of INT2 is overall exergonic by Δ*G*_298_ of 12 kcal mol^−1^, yielding a free “PP radical,” [(–_β_CH_2_–_α_C^•^(_γ_CH_3_)–)] (rINT), which can be subsequently attacked by another MnO_4_^−^ moiety via TS2a. The O-rebound pathway is viable only if INT2 remains; the transfer of its OH moiety proceeds via TS3 with a high Δ*G*^‡^_298_ of 33 kcal mol^−1^. Therefore, we suggest that free rINT is formed instead. Consequently, the oxo ligand from another MnO_4_^−^ ion abstracts an H-atom from the β–position of rINT to form an alkene, [–_β_CH = _α_C(R)–], within the PP polymer chain via TS2a (Δ*G*^‡^_298_ = 17 kcal mol^−1^), which is accompanied by a large release of Gibbs free energy (~37 kcal mol^−1^ with respect to rINT). It should be noted that the desaturation of alkanes generally involves a metal–OH intermediate to abstract the second H-atom from an alkane radical [47]. However, according to our calculated energetics, the second H-atom abstraction from the β-position of the radical site by another metal-oxo is overwhelmingly favorable. We have also investigated alternative pathways, such as the abstraction of a second H-atom from the α–position of the neighboring PP repeating unit via TS2b. The Δ*G*^‡^_298_ (TS2b) is 45 kcal mol^−1^, which is identical to the Δ*G*^‡^_298_ (rTS) value for the β, α-hydrogen atom shift. In other words, following the formation of a radical site, H-atom abstraction at the neighboring carbon atom is greatly facilitated. Thus, the most plausible mechanism for desaturation is successive H-atom abstraction at the α- and β-positions within the same repeating unit of PP. The potential energy surfaces (Schemes S1 and S2) for the desaturation reactions of PE and PVC also support this conclusion, despite some minor differences in the energetics arising from the different substituents.

## 4. Conclusions

This study demonstrated an effective mechanochemical method to activate inert PE, PP, and PVC through a solid-state reaction by ball milling with potassium permanganate. Our results indicated that the formation of alkene units in the polymer chain was evidence for a desaturation mechanism. Oxidation of the polymer by a sufficient amount of KMnO_4_ led to polymer breakdown and the formation of gaseous fuels such as methane, carbon monoxide, and hydrogen. Although at this moment it is not economically viable to use a quantitative amount of permanganate to generate coal gas from plastic waste (despite the fact that the produced Mn (II) oxide can be recycled back to KMnO_4_ for further oxidation), our system provides insights for the further development of metal-oxo catalytic systems for the large-scale oxidation of solid plastic waste, which may have a positive impact on environmental chemistry and petrochemistry.

## Figures and Tables

**Table 1:** Characteristics of the products recovered from the mechanochemical degradation of PE (0.50 g, 18 mmol), PP (0.50 g, 12 mmol), or PVC (0.50 g, 8 mmol) with using KMnO_4_ (1.2 molar equivalents: 21.6 mmol for PE; 14.4 mmol for PP; and 9.6 mmol for PVC) ^a^.

Entry	Substrate	Products (mmol)
1	Virgin PE	H_2_ (5.2 mmol), CH_4_ (2.7 mmol), CO (6.8 mmol), CO_3_^2−^ (16.2 mmol) (7.5%, 18.9%, and 45.0% of the carbon atom from polymer feed converted to CH_4_, CO, and CO_3_^2−^, respectively; 14.5% and 15.0% of the hydrogen atom from polymer feed converted to H_2_ and CH_4_, respectively.) ^b^
2	Virgin PP	H_2_ (5.6 mmol), CH_4_ (4.2 mmol), CO (5.9 mmol), CO_3_^2−^ (14.5 mmol) (11.7%, 16.4%, and 40.3% of the carbon atom from polymer feed converted to CH_4_, CO, and CO_3_^2−^, respectively; 15.6% and 23.3% of the hydrogen atom from polymer feed converted to H_2_ and CH_4_, respectively.) ^b^
3	Virgin PVC	H_2_ (3.5 mmol), CH_4_ (0.4 mmol), CO (8.9 mmol), CO_3_^2−^ (4.6 mmol), Cl^−^ (8.0 mmol) (2.5%, 55.6%, and 28.8% of the carbon atom from polymer feed converted to CH_4_, CO, and CO_3_^2−^, respectively; 29.2% and 6.7% of the hydrogen atom from polymer feed converted to H_2_ and CH_4_, respectively; 100% of the chlorine atom from polymer feed converted to Cl^−^.) ^b^
4	HDPE washing bottles	H_2_ (4.0 mmol), CH_4_ (2.0 mmol), CO (6.1 mmol), CO_3_^2^^−^ (18.9 mmol) (5.6%, 16.9%, and 52.5% of the carbon atom from polymer feed converted to CH_4_, CO, and CO_3_^2−^, respectively; 11.1% and 11.1% of the hydrogen atom from polymer feed converted to H_2_ and CH_4_, respectively.) ^b^
5	PP microwave boxes	H_2_ (5.0 mmol), CH_4_ (4.7 mmol), CO (5.4 mmol), CO_3_^2−^ (11.6 mmol) (13.1%, 15.0%, and 32.2% of the carbon atom from polymer feed converted to CH_4_, CO, and CO_3_^2−^, respectively; 13.9% and 26.1% of the hydrogen atom from polymer feed converted to H_2_ and CH_4_, respectively.) ^b^
6	PVC water piping	H_2_ (2.5 mmol), CH_4_ (0.4 mmol), CO (6.2 mmol), CO_3_^2−^ (6.6 mmol), Cl^−^ (8.0 mmol) (2.5%, 38.8%, and 41.3% of the carbon atom from polymer feed converted to CH_4_, CO, and CO_3_^2−^, respectively; 20.8% and 6.7% of the hydrogen atom from polymer feed converted to H_2_ and CH_4_, respectively; 100% of the chlorine atom from polymer feed converted to Cl^−^.) ^b^

^a^ Reaction conditions: Ball-milling of the polymer in the presence of KMnO_4_ at 800 rpm for 48 h under vacuum. ^b^ Percentage conversion (%) = (moles of hydrogen or carbon atom in fuel gas/moles of hydrogen or carbon atom in polymer feed) × 100.

## Data Availability

The data that support the findings of this study are available from the corresponding author, upon reasonable request.

## Supplementary Materials

The following are available online at https://www.mdpi.com/article/10.3390/polym13213672/s1, Figure S1: GC-TCD chromatograms of the gaseous products generated from the mechanochemical solid-state reaction of virgin PE (0.50 g, 18 mmol), PP (0.50 g, 12 mmol), or PVC (0.50 g, 8 mmol) with KMnO_4_ (1.2 molar equivalents: 21.6 mmol for PE; 14.4 mmol for PP; and 9.6 mmol for PVC) at a ball milling speed of 800 rpm for 12 h under vacuum conditions showing peaks for (i) hydrogen, (ii) nitrogen, (iii) oxygen, (iv) methane, and (v) carbon monoxide, Figure S2: (a–c) FTIR characterization, (d–f) XPS analysis, and (g–i) SEM images of virgin PE, **PE****[Ox]_vac_**, and **PE****[Ox]_vac/acid_**, Figure S3: (a–c) FTIR characterization, (d–f) XPS analysis, and (g–i) SEM images of virgin PP, **PVC****[Ox]_vac_**, and **PVC****[Ox]_vac/acid_**, Table S1: XPS analysis of PE, **PE****[Ox]_vac_**, **PE****[Ox]_vac/acid_**, PP, **PP****[Ox]_vac_**, **PP****[Ox]_vac/acid_**, PVC, **PVC****[Ox]_vac_**, **PVC****[Ox]_vac/acid_**, Scheme S1: (Top) Potential energy surface for the oxidation of PE by MnO_4_^−^ at the B3LYP/LanL2DZ level of theory for Mn and the B3LYP/6-31++G(d,p) level of theory for non-metal atoms. All energies are in kcal/mol and relative to those of the free reactants. (Bottom) Structures of intermediates (INTs) and transition states (TSs); the unit of bond distance is Å, Scheme S2: (Top) Potential energy surface for the oxidation of PVC by MnO_4_^−^ at the B3LYP/LanL2DZ level of theory for Mn and the B3LYP/6-31++G(d,p) level of theory for non-metal atoms. All energies are in kcal/mol and relative to those of the free reactants. (Bottom) Structures of intermediates (INTs) and transition states (TSs); the unit of bond distance is Å, Video S1: Burning the gases collected from ball milling of real plastic waste (HDPE washing bottle, 18.0 mmol) with KMnO_4_ (21.6 mmol) under vacuum at 25 °C for 48 h.
