# Supplementary material for: Waste-to-Energy: Production of Fuel Gases from Plastic Wastes
Source: Polymers (Basel). 2021 Oct 25;13(21):3672. doi: 10.3390/polym13213672 (PMC8588166; doi:10.3390/polym13213672)
Supplement: Supplementary file 1 [file polymers-13-03672-s001.zip › polymers-1390335-supplementary.pdf]

Supplementary information

Waste-to-energy: Production of Fuel Gases from Plastic Wastes

Cheuk-Fai Chow [1,]*, Chow-Shing Lam [2], Kai-Chung Lau [2,]* and Cheng-Bin Gong [3]

[1]*Department of Science and Environmental Studies, The Education University of Hong Kong, 10 Lo Ping Road, Tai Po Hong Kong SAR, China.*

[2]*Department of Chemistry, City University of Hong Kong, Kowloon Tong, Hong Kong SAR, China.*

[3]*The Key Laboratory of Applied Chemistry of Chongqing Municipality, College of Chemistry and Chemical Engineering, Southwest University, Chongqing, 400715, China.*

*Prof. Cheuk-Fai Chow. E-mail: cfchow@eduhk.hk; Tel: (+852) 29487671; Fax: (+852) 29487676.

Video S1. Burning the gases collected from ball milling of real plastic waste (HDPE washing bottle, 18.0 mmol) with $KMnO_4$ (21.6 mmol) under vacuum at 298 K for 48 h.

https://drive.google.com/open?id=1r2WF_zwaA7aZpsi5cglS84pvfWnclZbd

Table S1. XPS analysis of PE, PE[Ox]$_{vac}$, PE[Ox]$_{vac/acid}$, PP, PP[Ox]$_{vac}$, PP[Ox]$_{vac/acid}$, PVC, PVC[Ox]$_{vac}$, PVC[Ox]$_{vac/acid}$.

Samples	Overall Percentage (atom%)				Elemental composition (atom%) C1s			
	C_{1s}	O_{1s}	Cl_{2p}	Others	C=C	C–C	C–O /C–Cl	C=O
Virgin PE	100	0	0	0	0	100	0	0
**PE[Ox]$_{vac}$**	95.3	3.1	0	1.6	13.4	76.3	8.7	1.6
**PE[Ox]$_{vac/acid}$**	93.2	4.8	0	2.0	21.3	62.9	14.4	1.4
Virgin PP	100	0	0	0	0	100	0	0
**PP[Ox]$_{vac}$**	94.3	3.9	0	1.8	12.0	76.1	11.9	0
**PP[Ox]$_{vac/acid}$**	93.9	3.7	0	2.4	15.6	73.3	10.4	0.7
Virgin PVC	66.6	0	33.3	0	0	59.8	40.2	0
**PVC[Ox]$_{vac}$**	76.4	7.8	14.8	1.0	28.7	35.6	27.0	8.7
**PVC[Ox]$_{vac/acid}$**	80.0	6.7	13.1	0.2	37.6	33.1	20.2	9.1

PE (0.50 g, 18 mmol), PP (0.50 g, 12 mmol), or PVC (0.50 g, 8 mmol) by $KMnO_4$ (0.2 molar equivalents: 3.6 mmol for PE; 2.4 mmol for PP; and 1.6 mmol for PVC). All reactions were carried out under vacuum using solid-state ball milling at 800 rpm for 48 h.

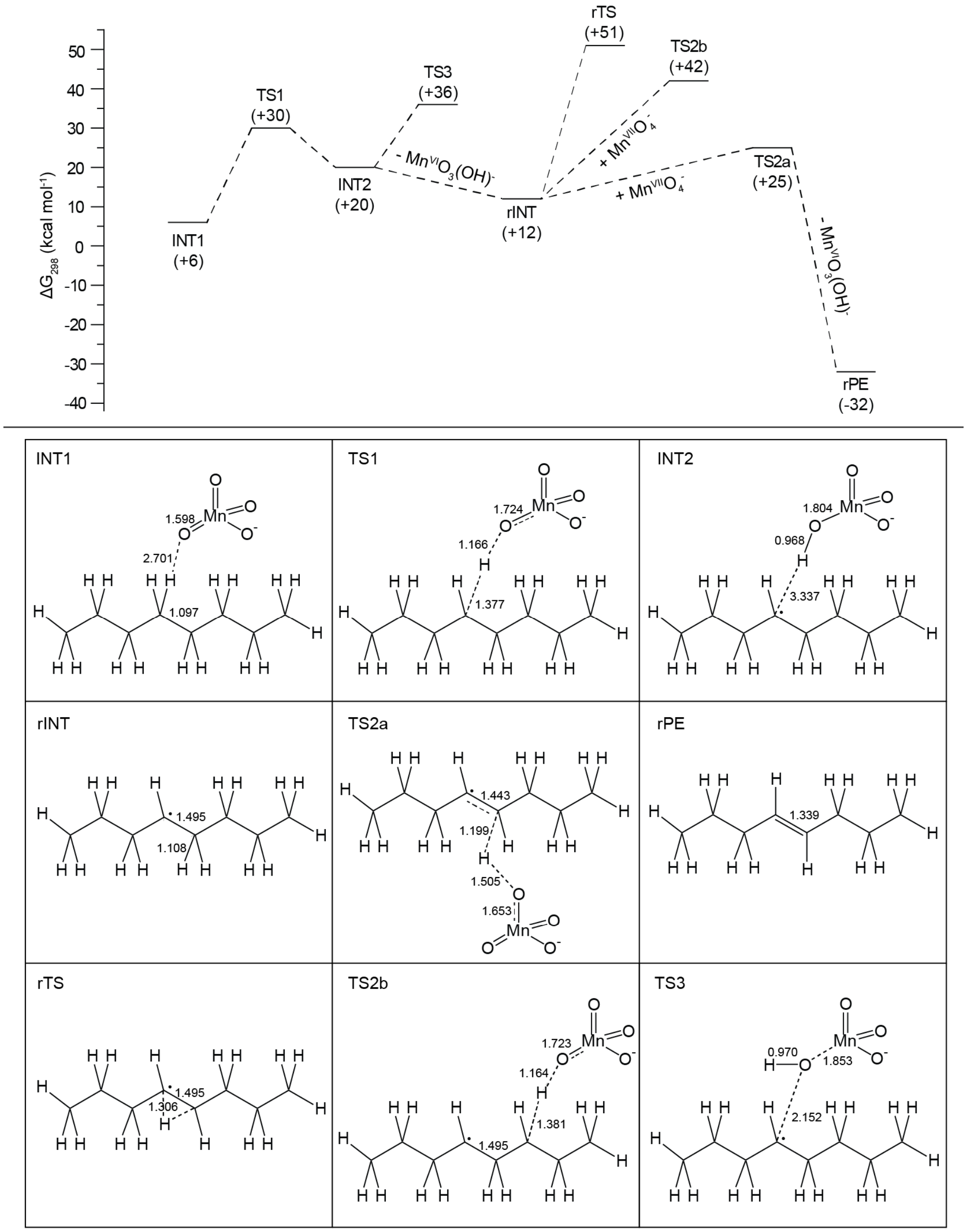

Scheme S1. (**Top**) Potential energy surface for the oxidation of PE by MnO_4^- at the B3LYP/LanL2DZ level of theory for Mn and the B3LYP/6-31++G(d,p) level of theory for non-metal atoms. All energies are in kcal/mol and relative to those of the free reactants. (**Bottom**) Structures of intermediates (INTs) and transition states (TSs); the unit of bond distances is Å.

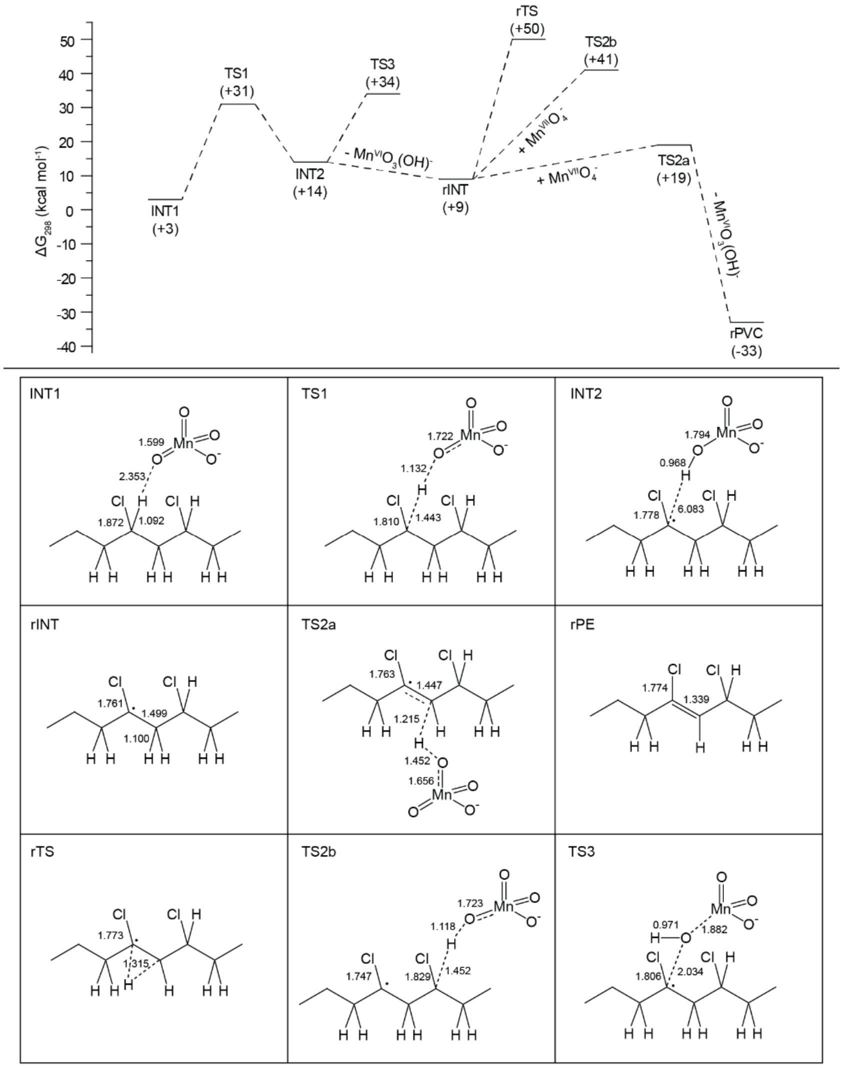

Scheme S2. (**Top**) Potential energy surface for the oxidation of PVC by MnO_4^- at the B3LYP/LanL2DZ level of theory for Mn and the B3LYP/6-31++G(d,p) level of theory for non-metal atoms. All energies are in kcal/mol and relative to those of the free reactants. (**Bottom**) Structures of intermediates (INTs) and transition states (TSs); the unit of bond distances is Å.

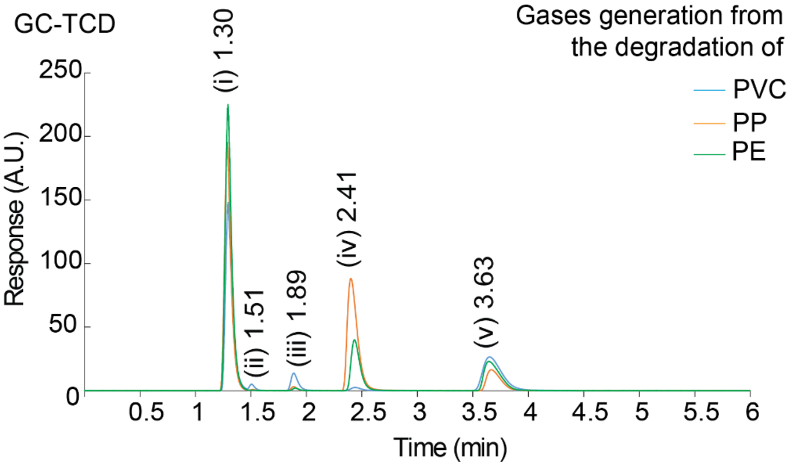

Figure S1. GC-TCD chromatograms of the gaseous products generated from the mechanochemical solid-state reaction of virgin PE (0.50 g, 18 mmol), PP (0.50 g, 12 mmol), or PVC (0.50 g, 8 mmol) with $KMnO_4$ (1.2 molar equivalents: 21.6 mmol for PE; 14.4 mmol for PP; and 9.6 mmol for PVC) at

a ball milling speed of 800 rpm for 48 h under vacuum conditions showing peaks for (i) hydrogen, (ii) nitrogen, (iii) oxygen, (iv) methane, and (v) carbon monoxide.

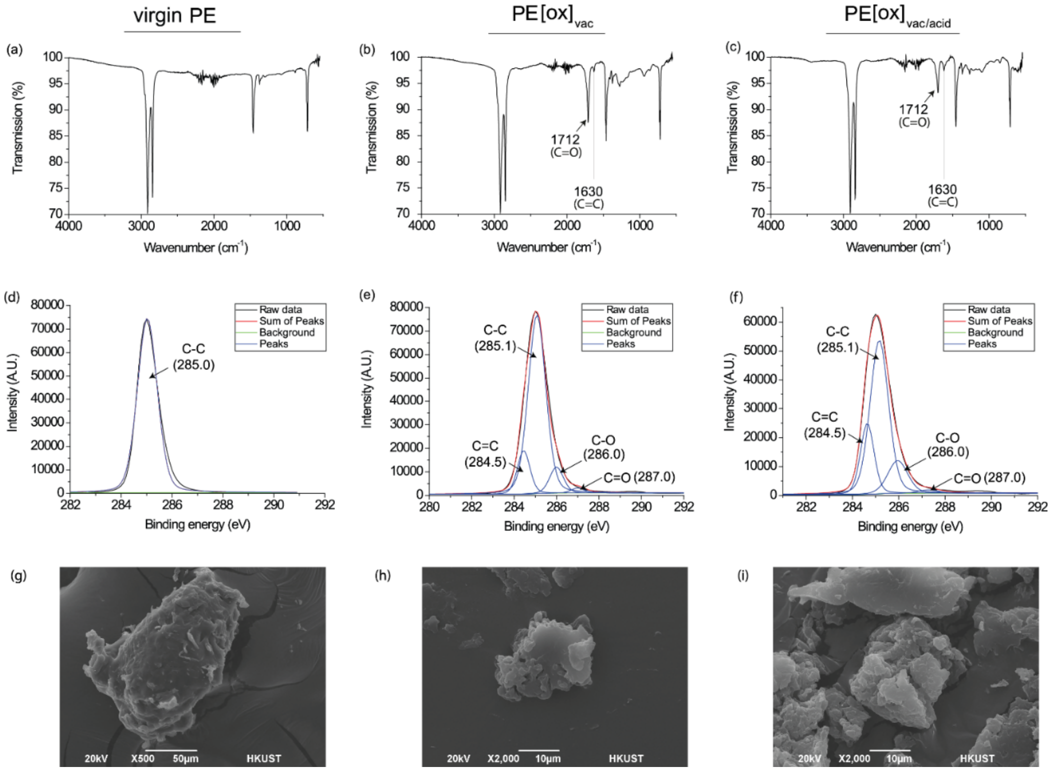

Figure S2. (a–c) FTIR characterization, (d–f) XPS analysis, and (g–i) SEM images of virgin PE, **PE[Ox]vac**, and **PE[Ox]vac/acid**.

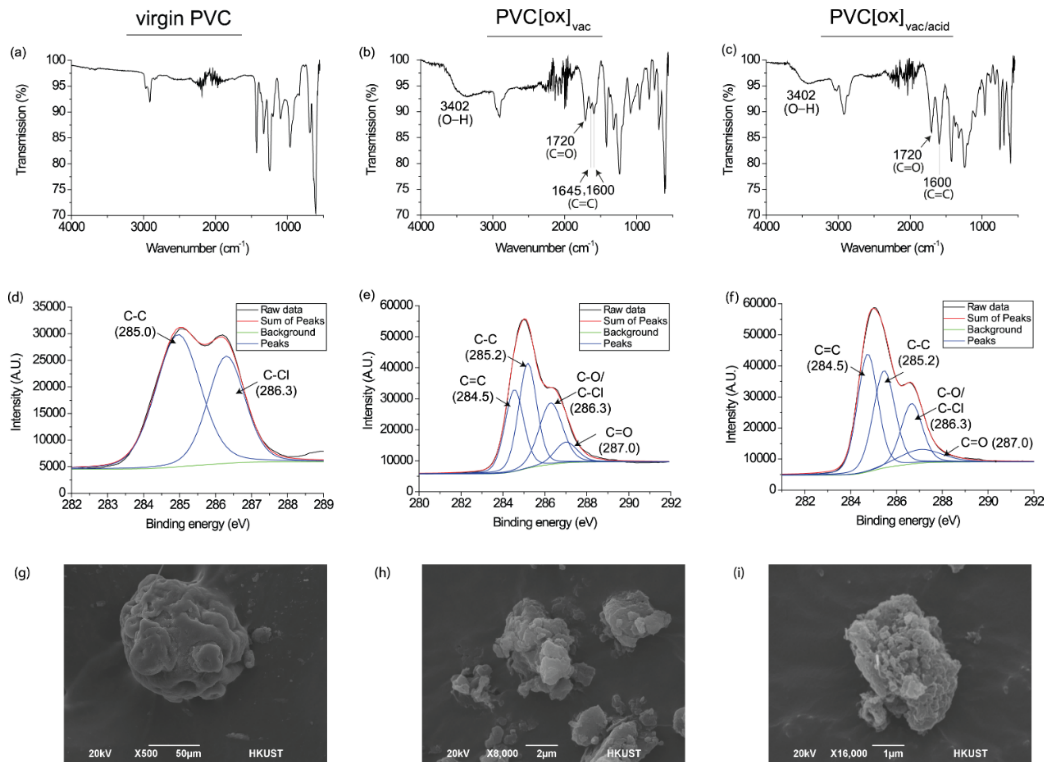

Figure S3. (a–c) FTIR characterization, (d–f) XPS analysis, and (g–i) SEM images of virgin PP, **PVC[Ox]vac**, and **PVC[Ox]vac/acid**. .
